# Wildlife forensics in battle against veneration frauds in Uttarakhand, India: identification of protected Indian monitor lizard in items available in the local market under the name of Hatha Jodi

**DOI:** 10.1080/23802359.2018.1501284

**Published:** 2018-08-17

**Authors:** Ankita Rajpoot, Ved Prakash Kumar, Archana Bahuguna, Tribhuwan Singh, Sunny Joshi, Dhyanendra Kumar

**Affiliations:** a Molecular Systematic Laboratory, Zoological Survey of India, NRC, Dehradun, India;; b MAATY Biodiversity Conservation and Societal Research Organization, Dehradun, India;; c Wildlife Institute of India, Dehradun, India;; d Veer Kunwar Singh University, Arrah, India

**Keywords:** Hatha Jodi, Indian monitor lizard, DNA forensics, Mitochondrial genes and Conservation

## Abstract

Certain articles of worship are commonly sold in Uttarakhand, India by the name Hatha Jodi, a root of a rare plant found only in a few parts of central India. The present work provides genetic proof that the Hatha Jodi sold in three local markets of Uttarakhand contained material from the *Varanus* species, species protected under the Indian Wildlife (Protection) Act, 1972. A total of eight samples were bought, two each from the local markets in Haridwar and Rishikesh, three from Dehradun and one from an online source (Amazon). The initial inspection confirmed that two of the samples were made of plastic material. Therefore only the other six samples were subjected to DNA analysis. DNA sequences were successfully obtained and matched with reference sequences available in NCBI Genbank database through BLAST search tool for species identification. All the six samples matched 100% with the Indian monitor lizard. The findings indicate how commercialization and the wildlife trade are playing a role in decline of the population of the Indian monitor lizard. If strong protection measures are not taken as soon as possible, the Indian monitor lizards will go Extinct very soon. Therefore, we suggest that the Government and Wildlife enforcement agencies take serious action against the illegal articles available in the local markets of Uttarakhand under the name Hatha Jodi. Further, the government needs to take legal action against offenders in other states in which the product is available for sale.

## Introduction

The term ‘wildlife crime’ is defined as the trafficking of live and dead specimens of animals, plants and their derivatives. Wildlife crime involves illegal poaching and exploitation of wild animals for meat, trophies, traditional medicines, as articles for worship, ornaments, etc. Currently, wildlife crime is the most urgent threat faced by many species and is resulting in their decline and extinction (Linacre et al. 2011). However, fake wildlife products are being sold in the market for economic gain although it is a consumer’s right to seek details of the products he or she buys online. There is hardly any control or assurance about the products sold in local markets. Thus, nowadays a number of fake products are being sold fraudulently in the local markets and shopping websites to the consumers (Linacre et al. 2011; Coghlan et al. 2012; Mukesh et al. 2017). Selling certain types of meat and plant products is governed by various laws, depending on the countries’ faunal resources and status of the species involved. Sometimes meat and plant parts obtained from the protected species are sold under the name of the species and/or products which can be sold legally. Thereby serious wildlife crimes are committed in disguise. These become unsustainable when there is trading on a commercial scale (Mukesh et al. 2017).

Recently the print and electronic media highlighted how '*Varanus’* genital parts are being sold in the local markets of India and through the websites under the name Hatha Jodi, which refers to the material obtained from the root of a plant (Scroll.in 2017; News. nationalgeographic.com 2017; Hindustantimes.com 2017; Worldanimalprotection.org.in 2017). The plant is very rare and the root is often described as resembling human arms with clenched fists. The plant is generally found in central India and is therefore called the “Heart of India”. Hatha Jodi is used by humans as a good luck charm and for financial benefits (Pellegrini 2015). Because the plant is rare and there is a great demand in the Indian market, sellers look for similar products. Morphologically, the Hatha Jodi root is similar to the genital parts of the *Varanus* species, and it is very difficult to distinguish them. So the people involved in the business have a substitute for Hatha Jodi in *Varanus* parts.

India is home to four species of monitor lizard—the Bengal monitor (*Varanus bengalensis*), the Water monitor (*Varanus salvator*), the Yellow monitor (*Varanus flavescens*) and the Desert monitor (*Varanus griseus*) (Rao 1994). The Bengal monitor is widely distributed throughout India, the Desert monitor is present in Rajasthan and Punjab, the Yellow monitor occurs in the Gangetic plain and is most common in Uttar Pradesh and Bihar, and the Water monitor is found in Orissa, Bengal and eastern India (Rao 1994). The possession and trade of these species are prohibited under the Indian Wildlife (Protection) Act, 1972, and they are listed as "Schedule-I" species. These species are classified as "Least Concern (LS)" by the International Union of Conservation Nature (IUCN) (IUCN Red List 2010). Internationally, all four Indian monitor lizards were listed in the "Appendix-I" of the Convention on International Trade in Endangered Species of Wild Fauna and Flora (CITES), which prohibits international commercial trade in them (CITES 2017).

For a few decades, *Varanus* species are being hunted for their skin. The leather is used for making percussion instruments,the meat and body parts of *Varanus* species are believed to have aphrodisiac properties, and these have been used in traditional medicine (Phillips and Packard 1994; Shine et al. 1996, 1998; Pernetta 2009; Scheffers et al. 2012; Welton et al. 2013). Now the reproductive organs of these animals are used in the articles of worship in the place of a plant’s root. Therefore the entire monitor lizard is utilized by poachers for economic benefits. Unfortunately, we have no data regarding their population status. Thus it is difficult to make an authentic statement about their population trends. But on the basis of the reports about *Varanus* poaching and seizures of *Varanus* parts, we can say that their populations are declining rapidly. A conservation action plan for *Varanus* requires research into the populations of the species through genetic structure, phylogeography, and landscape ecology.

The aim of the present study was to reconfirm the reports in the media and other sources in Uttarakhand by using DNA forensics. Identification of species was based on molecular genetics and has been used to investigate the illegal hunting of vertebrates (Wu et al. 2005; Rajpoot et al. 2016) and the trade in protected species and species derivatives (Baker and Palumbi 1994; Kumar et al. 2014). Genetics-based species detection attempts to match an unidentified sample with a reliably known reference sample by comparing sequences of the mitochondrial genes.

Mitochondrial Cyt b, 12S rRNA, and 16S rRNA are the genetic markers used most frequently for the species discrimination in degraded samples (Karlsson and Holmlund 2007). Short regions of universal mitochondrial DNA have been developed for various animal taxa and used to assign species in different challenging forensic samples, which may contain degraded DNA, including hair shafts, bones, feathers, and meat products (Parson et al. 2000; Kocher et al. 1989; D'Amato et al. 2013). Wildlife DNA forensics plays a significant role in identifying the species in the illegal trade of endangered wildlife (Iyengar 2014) and in the detection of species in mislabelled products and fraud (pooja items, food products) (Galimberti et al. 2013).

## Materials and methods

### Case history

**Figure 1.**
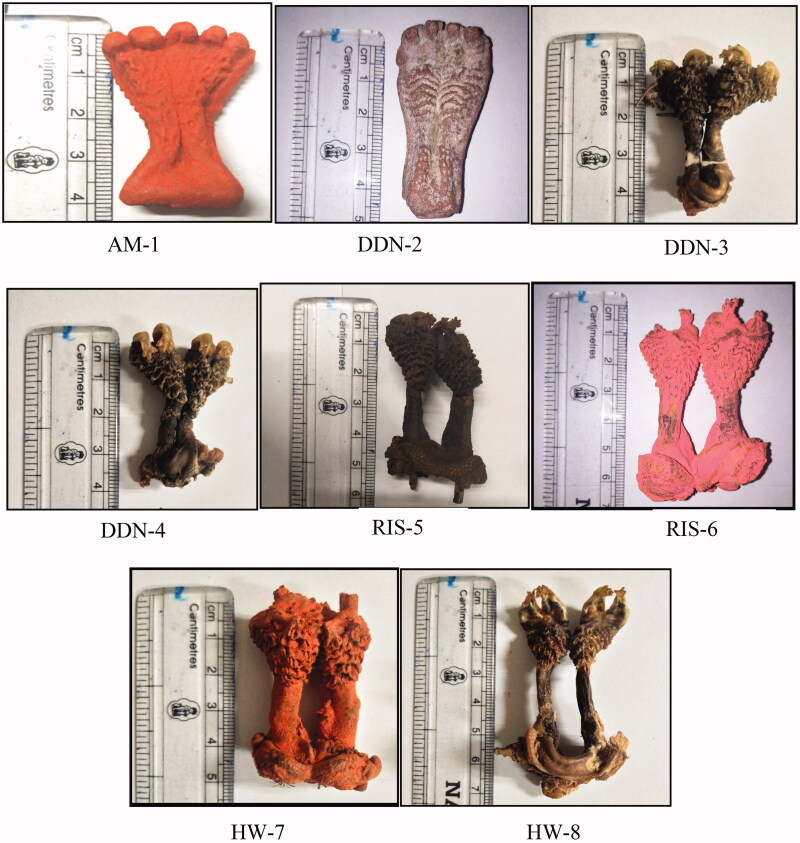
Samples purchase from online website and local market in Uttarakhand.

During our field surveys (2016–2017) in Uttarakhand, under the ongoing project, we had seen that Hatha Jodi was being sold in various shops which were selling articles for worship in Haridwar, Rishikesh, and Dehradun. We conducted interviews with the shopkeepers and enquired about this product. They said that it was a plant material, and was used in poojas, especially during Deepavali, and a few people were also using it in tantric poojas. Recently we saw reports in the print media that wildlife enforcement agencies had seized similar articles as these were suspected to be the animal parts. Thereafter, we had the noted such illegal activities in Uttarakhand also. Having already seen items like Hatha Jodi in Haridwar, Rishikesh, and Dehradun, we surveyed the same places again (December 2017 to January 2018) to confirm whether the items involved were really plant materials or were animal parts. We bought eight (*n* = 8) Hatha Jodi samples from an online source (Amazon, AM 1), from Dehradun (DDN 2 to DDN 4), Rishikesh (RIS 5 and RIS 6), and Haridwar (HW 7 and HW 8) (Table 1 and Figure 1). Thereafter we tested the purchased samples in a molecular laboratory to confirm our suspicions.

**Table 1.** Details of samples purchased from online website and local market of Uttarakhand.

Sample	Origin	Name of Item selling in marketand online website	No. Items	Cost (In Indian rupees)	Source	Visual inspection
AM-1	Amazon	Hatha Jodi	1	1200/-	Online	Plastic material
DDN-2	Dehradun	Hatha Jodi	1	50/-	Local market	Plastic material
DDN-3	Dehradun	Hatha Jodi	1	1100/-	Local market	Tissue
DDN-4	Dehradun	Hatha Jodi	1	1100/-	Local market	Tissue
RIS-5	Rishikesh	Hatha Jodi	1	1200/-	Local market	Tissue
RIS-6	Rishikesh	Hatha Jodi	1	1800/-	Local market	Tissue
HW-7	Haridwar	Hatha Jodi	1	1000/-	Local market	Tissue
HW-8	Haridwar	Hatha Jodi	1	900/-	Local market	Tissue

### DNA extraction

The possibility of human contamination of the samples was high. So we cleaned the samples first with ethanol and 10× phosphate-buffered saline (PBS) two times. Interestingly, after two samples (AM 1 and DDN 2) were washed, what appeared to be plastic materials were noted. These samples were burnt to confirm that they were plastics, and both samples melted. Therefore these two samples were not used in the present study, whereas the other six samples were used. The outer surface (0.5 mm) of the six samples was removed using a surgical blade to remove surface contaminants, and the surface was washed again with 10 × PBS. A small section of the basal part of these samples was cut, chopped and transferred to a 2µl Eppendorf tube (Axygen, Union City, California). DNA extraction was attempted using a QIAGEN DNeasy Tissue Kit (QIAGEN, Hilden, Germany) according to the manufacturer’s instructions. To determine the quality and concentration of the DNA obtained, the samples were subjected to gel electrophoresis on a 0.8% agarose gel in 1 × TAE buffer and DNA quantified with a UV spectrophotometer (Amersham Pharmacia, Centennial Avenue, Piscataway, New Jersey, USA). All measures were taken to avoid and monitor the contamination.A negative control was included during the extraction of DNA.

### PCR amplification

A common PCR condition was used for two mitochondrial DNA fragments, Cyt b (Verma and Singh 2003) and 16S rRNA (Mitchell et al. 1993). Approximately 50 ng of the total DNA was used as a template for PCR in a final volume of 25 μl, containing 10 mM of 2.5 μl PCR buffer, 1.5 mM MgCl_2_, 0.1 mM dNTP, 0.5 μM of each primer, and 1.25 U of Taq DNA polymerase. All the PCR reactions were carried out in a Thermal Cycler (Quanta Biotech) using the protocol which was published previously by Rajpoot et al. (2016). The products were subjected to gel electrophoresis on a 1.5% agarose gel in 1 × TAE buffer to determine the concentration and quality of the amplicon obtained.

### DNA sequencing

The amplified PCR products were purified using Exo-SAP to remove the residual oligonucleotides and dNTPs prior to the sequencing reaction. The forward primer of Cyt b and 16S rRNA was used independently for the sequencing reactions using the Big Dye^®^ Terminator v3.1 Cycle Sequencing Kit and to generate the sequence. The products were purified by a standard ethanol precipitation method and were sequenced commercially. The sequences reported in this paper have been submitted to NCBI’s GenBank, and the accession numbers are awaited.

#### Data analysis

The sequences obtained were visualized and edited using Chromas 2.6.4 (Technelysium Pty Ltd., South Brisbane, Australia) (www.technelysium.com.us). Multiple sequence alignments were performed using the CLUSTAL W algorithm implemented in BioEdit version 7.0.5.3 (Hall 1999). The tree topology was generated using the neighbor-joining method (Saitou and Nei 1987) with 1000 bootstrap values (Felsenstein 1985) for both mitochondrial genes. The Mega v7.0 software package was used for the implementation (Kumar et al. 2016). Aligned columns with gaps or missing data were eliminated. The sequences obtained from the samples were compared with the reference sequences available at GenBank using the search tool BLAST of NCBI (http://blast.ncbi.nlm.nih.gov/). All the sequences were compared with our reference sequences of the four Indian monitor lizards available in GenBank.

## Results and discussion

**Figure 2.**
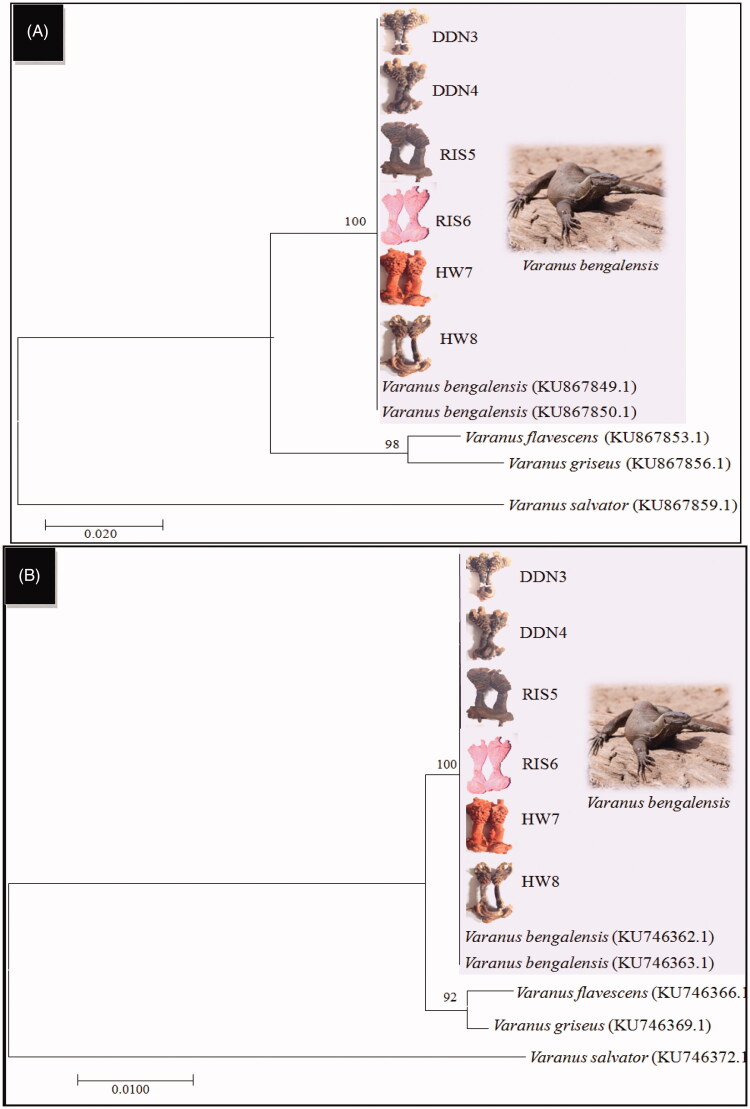
Cytochrome b (A) and 16s ribosomal RNA (B) sequences based Neighbor-Joining (NJ) tree of purchased samples with previously published references sequences (Rajpoot et al., 2016) of four Indian Varanus species.

The samples used (*n* = 6 samples) yielded genomic DNA of good quality. The DNA concentration ranged from 50 ng to 60 ng in four samples and from 25 ng to 30 ng in the other two samples. Both mitochondrial genes (Cyt b and 16S rRNA) were successfully amplified in all the samples using PCR. The mtDNA genes sequences obtained from all the samples were good, and the sequence lengths were 396 bp (Cyt b) and 471 bp (16S rRNA). Initially, we matched these sequences with the data published by us previously (Rajpoot et al. 2016) where all the six purchase samples showed 100% similarity with Bengal monitor lizard (Table 2 and 3). In our previous work, we established a database of the four species of Indian monitor to solve wildlife forensic cases using the FINS (Forensically Informative Nucleotide Sequences) approach. Furthermore, the sequences obtained of both genes were submitted as independent entries in a BLAST search for cross–checking, and similar results were obtained (Table 4). The neighbor-joining tree from Cyt b and 16S rRNA also showed that the samples were obtained clustered with the Bengal monitor lizard with a 100% bootstrap value (Figure 2A and 2B). Thus, it is clear that the plant material purchased (Hatha Jodi) was originated from Bengal monitor lizards, which is a legally protected species. Poaching and the trade of their biological parts are prohibited.

**Table 2.**
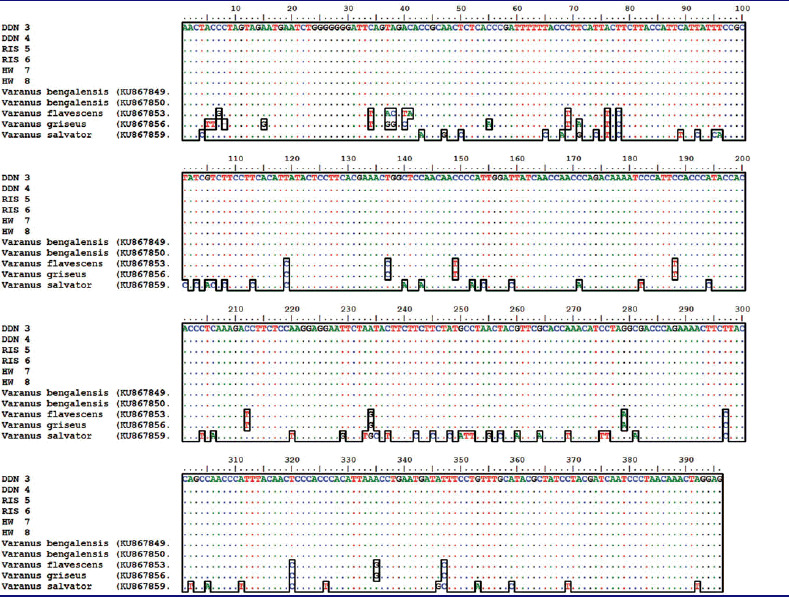
Cytochrome b sequences based multiple sequences alignment of purchased samples (Hatha Jodi) from local market of Uttarakhand with our previously published reference sequences (Rajpoot et. al., 2016) of four Indian Varanus species.

**Table 3.**
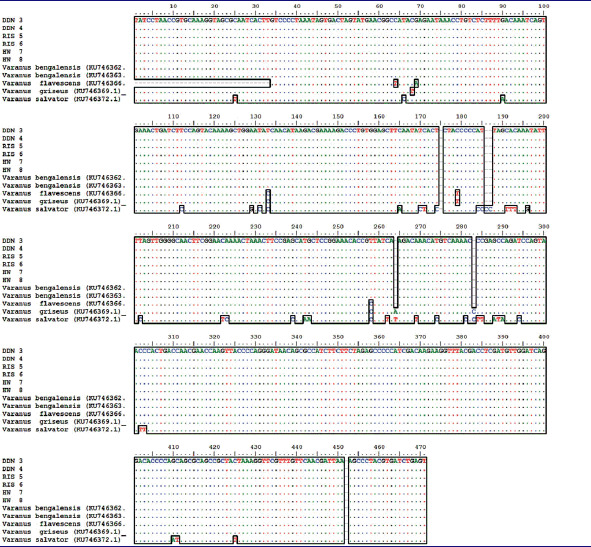
16S rRNA sequences based multiple sequences alignment of purchased samples (Hatha Jodi) from local market of Uttarakhand with our previously published reference sequences (Rajpoot et. al., 2016) of four Indian Varanus species.

**Table 4.** Species similarity and percent scores generated using BLAST and CLUSTALW (with references samples) by comparison of Cyt b (396 bp) and 16S rRNA (470 bp) query sequences of the present study samples.

Species similarity with BLAST result	Species similarity with references sequences	BLAST Cyt b/16S rRNA (%)	Clustal W with referencessequences (%)
*Varanus bengalensis*	*Varanus bengalensis*	100/100	100/100
*Varanus salvator*	*Varanus salvator*	97/98	96/98
*Varanus griseus*	*Varanus griseus*	97/99	95/99
*Varanus flavescens*	*Varanus flavescens*	98/99	96/99

The Bengal monitor lizard is widely distributed all over India. It has the ability to cope with a broad range of environments, from deserts to rainforests. In the last few decades, the Bengal monitor population has been declining due to a high demand in the black market for the skin, meat, bones, etc. of the species (Traffic Post 2013). Now there is a thriving trade of the reproductive organs. In India, tantric practitioners strongly believe that, if appropriately energized by a guru, Hatha Jodi can change a person’s life by bringing immense wealth, happiness, enhanced sex power, luck in gambling, and prosperity in business (indianetzone.com 2013). Unfortunately, in this developing world, people tend to fall back on such black magic remedies. The genital organ of this poor animal will not help you to become rich. Monitor lizards are being hunted due to lack of awareness, superstition of society, and cruelty.

DNA-based techniques have been demonstrated to be robust and sufficiently advanced to solve the wildlife offences. They are used to reveal the composition of spiritual items (items used in poojas and tantra kriya) about which fraudulent claims are made, adulterated food, and endangered plants and animals in the illegal trade. The use of DNA evidence in forensic investigations in the last two decades has played a crucial role in fighting against the poaching of endangered and protected species and in preventing cruelty towards animals. The continued development of a standardized set of protocols for wildlife forensics will further enhance the capacity of law enforcement officials to protect and conserve animals in the wild.

However, the present study is thought-provoking and provides insights into preventing wildlife offences involving Indian monitors in Uttarakhand. This study shows how the Indian monitor lizard is getting Extinct due to the commercialization of the wildlife trade. If the poaching of Indian monitor lizards and the trade in these species are not controlled, they will be Extinct very soon. Therefore, it is suggested that the Indian government and wildlife enforcement agencies should take serious action against Hatha Jodi, an illegal article, available in Uttarakhand markets. Further, search operations may be conducted in local markets in other states where it may be available. However, that this study will help wildlife conservationists and policymakers generate strong legislation and policy to protect Indian monitor lizards as soon as possible.
